# Modified Frailty Index Predicts Postoperative Complications following Panniculectomy in the Elderly

**DOI:** 10.1097/GOX.0000000000002987

**Published:** 2020-07-21

**Authors:** Jasmine Lee, Allyson R. Alfonso, Rami S. Kantar, Gustave K. Diep, Zoe P. Berman, Elie P. Ramly, David A. Daar, Jamie P. Levine, Daniel J. Ceradini

**Affiliations:** From the * Hansjörg Wyss Department of Plastic Surgery, NYU Langone Health, New York, N.Y.; † The University of Maryland Medical System/Shock Trauma Center, Baltimore, Md.

## Abstract

Due to the high complication rate of panniculectomies, preoperative risk stratification is imperative. This study aimed to assess the predictive value of the 5-item modified frailty index (mFI-5) for postoperative complications in the elderly following panniculectomy.

**Methods:**

A retrospective cohort study of the American College of Surgeons National Surgical Quality Improvement Program database for patients over the age of 65 years who underwent a panniculectomy between 2010 and 2015 was conducted. The mFI-5 score was calculated for each patient based on the presence of diabetes, hypertension, congestive heart failure, chronic obstructive pulmonary disease, and dependent functional status, and an mFI-5 score of 2 was used as a cutoff. Multivariate logistic and linear regression analysis was used to determine the validity of the mFI-5 as a predictor of postoperative complications.

**Results:**

A total of 575 patients were analyzed. Patients with an mFI-5 score of 2 or more (421; 73.2%) had significantly higher rates of wound complications (19.5% versus 12.8%; *P* = 0.03), overall complications (33.8% versus 19.5%; *P* < 0.001), and significantly longer hospital length of stay (3.6±5.0 versus 1.9±3.0; *P* < 0.001). mFI-5 score of 2 or more was an independent risk factor for wound complications (odds ratio, 1.26; 95% confidence interval, 1.08–2.20; *P* = 0.04) and overall complications (odds ratio, 1.34; 95% confidence interval, 1.09–2.15; *P* = 0.02).

**Conclusions:**

Frailty, as measured by the mFI-5, holds a predictive value regarding outcomes of wound complications and overall complications in elderly patients after panniculectomy. The mFI-5 score can be used to identify high-risk patients before surgery.

## INTRODUCTION

Abdominoplastic surgeries are among the most common surgical procedures performed by plastic surgeons in the United States, with 157,492 procedures performed in 2018.^1^ The rising popularity of bariatric surgery with subsequent successful weight loss contributes to this trend. It is estimated that 228,000 bariatric procedures were performed in 2017, and up to 74% of patients desire subsequent body contouring procedures post-bariatric surgery, including panniculectomy.^2,3^ Despite its popularity, panniculectomy is associated with high overall complication rates ranging from 23% to 44%.^4,5^ Complications most commonly include seroma, hematoma, tissue necrosis, and wound dehiscence. Although most complications can be managed conservatively, postoperative morbidity remains a concern.^6^

Due to the high complication rate of panniculectomies, risk stratification of patients before surgery is important. In particular, a patient after bariatric surgery may often have nutritional deficiencies, residual medical comorbidities, and a complex body habitus, which may negatively affect postoperative outcomes.^7^ Several studies have identified possible risk factors for post-panniculectomy complications, including preoperative body mass index (BMI), mass of the resected pannus, and hypertension.^6,8^ However, many of these risk factors have not been shown to be predictive across different populations.^8,9^ This indicates the need for a more comprehensive approach to risk stratification.

Frailty, or the measure of physiologic reserve and resistance to stressors, has been recognized as a predictor of healthcare outcomes.^10^ The modified frailty index (mFI-5) is a 5-factor index designed to stratify patients based on the level of frailty and has been shown to be an effective predictor of mortality and postoperative complications in several subspecialties, including plastic surgery.^10,11^ The mFI-5 comprises the following variables: hypertension requiring medication, diabetes mellitus, chronic obstructive pulmonary disease (COPD) or a diagnosis of pneumonia within 30 days, functional status (independent, partially dependent, or fully dependent), and congestive heart failure (CHF).^11^ These comorbidities have been identified as contributing factors to increased frailty, which predisposes patients to adverse outcomes following invasive surgery. The aim of this study was to evaluate the efficacy of the 5-factor mFI as a predictor of postoperative complications in patients who have undergone panniculectomy.

## METHODS

### Database and Patient Population

For the purpose of this study, the Participant Use Files database from the years 2010–2015 was reviewed. The database is compliant with the Health Insurance Portability and Accountability Act and is exempt from institutional review board (IRB) review. The American College of Surgeons National Surgical Quality Improvement Program (ACS-NSQIP) is a prospective, risk-adjusted, outcomes-based registry that records demographic, perioperative, and 30-day postoperative de-identified patient information.^12^ A retrospective review of the database was performed for the following current procedure terminology (CPT) code: 15830 [excision, excessive skin and subcutaneous tissue (includes lipectomy); abdomen, infraumbilical panniculectomy]. Cases recorded with a primary abdominoplasty CPT code [CPT 15847 (excision, excessive skin and subcutaneous tissue (includes lipectomy); abdomen (eg, abdominoplasty) (includes umbilical transposition and fascial plication)] were not included in our study.

### Study Design

As a retrospective cohort study, database review was performed for the selected CPT code, and cases with missing information on age, gender, weight, height, functional status, diabetes status, and history of COPD, CHF, or hypertension were excluded from the analysis. mFI-5 score was the risk factor of interest in this study, and only patients aged 65 years or older were included. We relied on the mFI-5 described by Subramaniam et al^11^ using the ACS-NSQIP database. These 5 factors include history of diabetes, hypertension, CHF, COPD, and dependent functional status. Each of these factors accounts for 1 point, and the minimum value for the mFI-5 is 0, while the maximum value is 5. mFI-5 score was analyzed as a binary categorical variable to compare groups with a low mFI-5 score of <2 with those with a high mFI-5 score of ≥2.^11,13,14^ Patient preoperative demographics, clinical factors, and medical comorbidities at the time of surgery were analyzed. Primary outcomes of the study included wound complication and overall complication. Wound complication was a composite outcome that consisted of superficial incisional surgical site infection, deep incisional surgical site infection, or wound dehiscence. Overall complication was a composite outcome that included developing any of the following: wound complication, reoperation, readmission, mortality, postoperative bleeding or transfusion requirement, deep venous thrombosis, pulmonary embolism, myocardial infarction, urinary tract infection, or sepsis. Secondary outcomes included operative time and hospital length of stay (LOS).

### Statistical Analysis

Continuous variables are reported as “mean ± SD,” while categorical variables are reported as frequencies and percentages within their corresponding groups. Univariate analysis was performed using χ^2^ or Fisher’s exact test (n < 10) for categorical variables and student’s *t* test for continuous variables. Potential confounders were controlled for using multivariate logistic and linear regression analyses. Variables included in our regression models included the following: mFI-5 score, age, BMI, gender, history of smoking, bleeding disorder, steroid use, American Society of Anesthesiology (ASA) classification, race, surgical team specialty, and wound classification. Statistical significance was defined as *P* value <0.05. All data analyses were performed using SPSS version 23.0 (IBM Corp., Armonk, N.Y.).

## RESULTS

Review of the database identified 7030 patients who underwent abdominal panniculectomy, and 575 of these were over the age of 65 years and eligible for inclusion in the study. Descriptive statistics of patient preoperative variables and characteristics are included in Table 1. Evaluation of primary outcomes showed that the rate of wound complications was 14.6%, while the rate of overall complications was 23.3%. The mean operative time (mean ± SD) was 156.7 ± 80.0 minutes, and the mean hospital LOS (mean ± SD) was 2.4 ± 3.7 days (Table 1).

**Table 1.** Descriptive Statistics of Preoperative Variables and Outcomes

Variable (N = 575)	n (%)
Frailty Index Score	
<2	421 (73.2)
≥2	154 (26.8)
Age, y (mean ± SD)	68.8 ± 3.6
BMI (mean ± SD)	34.2 ± 9.6
Men	103 (17.9)
Smoker	21 (3.7)
CHF	2 (0.3)
Hypertension	357 (62.1)
Diabetes	157 (27.3)
COPD	26 (4.5)
Bleeding disorder	13 (2.3)
Steroid use	11 (1.9)
Functional status	
Independent	555 (96.5)
Partial or totally dependent	20 (3.5)
ASA class	
Lower than 3	286 (49.7)
3 or higher	289 (50.3)
Race	
Asian	1 (0.2)
Black or African American	22 (3.8)
Native Hawaiian/Pacific Islander	3 (0.5)
White	549 (95.5)
Surgical specialty	
Plastic surgery	407 (70.8)
General surgery	168 (29.2)
Wound classification	
Clean	497 (86.4)
Clean/contaminated	40 (7.0)
Contaminated	27 (4.7)
Dirty/infected	11 (1.9)
Primary outcomes	
Wound complications	84 (14.6)
Complications	134 (23.3)
Secondary outcomes	
Operative time, min (mean ± SD)	156.7 ± 80.0
Hospital LOS, d (mean ± SD)	2.4 ± 3.7

DVT, deep venous thrombosis; MI, myocardial infarction; PE, pulmonary embolism; UTI, urinary tract infection.

When patients were stratified by mFI-5 score, 421 (73.2%) patients had a score <2 and 154 (26.8%) had a score of 2 or more. Patients with an mFI-5 score of 2 or more had a significantly higher BMI (39.8 ± 10.8 versus 32.1 ± 8.3; *P* < 0.001) and higher percentage of men (25.3% versus 15.2%; *P* = 0.01). There were significant differences between both groups in the ASA class (*P* < 0.001), racial distribution (*P* = 0.001), surgical team specialty distribution (*P* = 0.02), and distribution of wound classification (*P* = 0.03) (Table 2).

**Table 2.** Univariate Analysis of Preoperative Variables Stratified by Frailty Index

Variable (N = 7030)	Frailty Index Score		*P*
	<2 (n = 421), n (%)	≥2 (n = 154), n (%)	
Age (mean ± SD)	68.7 ± 3.5	69.1 ± 3.9	0.25
BMI (mean ± SD)	32.1 ± 8.3	39.8 ± 10.8	<0.001
Men	64 (15.2)	39 (25.3)	0.01
Smoker	17 (4.0)	4 (2.6)	0.62
Bleeding disorder	9 (2.1)	4 (2.6)	0.75
Steroid use	8 (1.9)	3 (1.9)	0.99
ASA class			
Lower than 3	256 (60.8)	30 (19.5)	<0.001
3 or higher	165 (39.2)	124 (80.5)	
Race			
Asian	0 (0)	1 (0.6)	0.01
Black or African American	10 (2.4)	12 (7.8)	
Native Hawaiian/Pacific Islander	2 (0.5)	1 (0.6)	
White	409 (97.1)	140 (90.9)	
Surgical specialty			
Plastic surgery	112 (26.6)	56 (36.4)	0.02
General surgery	309 (73.4)	98 (63.6)	
Wound classification			
Clean	372 (88.4)	125 (81.2)	0.03
Clean/contaminated	29 (6.9)	11 (7.1)	
Contaminated	15 (3.6)	12 (7.8)	
Dirty/infected	5 (1.2)	6 (3.9)	

Univariate analysis of primary and secondary outcomes stratified by mFI-5 score showed that patients with an mFI-5 score of 2 or more had significantly higher rates of wound complications (19.5% versus 12.8%; *P* = 0.03) and overall complications (33.8% versus 19.5%; *P* < 0.001), as well as significantly longer hospital LOS (3.6 ± 5.0 versus 1.9 ± 3.0; *P* < 0.001) (Table 3).

**Table 3.** Univariate Analysis of Outcomes Stratified by Frailty Index

Variable (N = 7030)	Frailty Index Score		*P*
	<2 (n = 421), n (%)	≥2 (n = 154), n (%)	
Primary outcomes			
Wound complications	54 (12.8)	30 (19.5)	0.03
Complications	82 (19.5)	52 (33.8)	<0.001
Secondary outcomes			
Operative time (mean ± SD)	160.0 ± 81.3	147.8 ± 75.7	0.09
Hospital LOS (mean ± SD)	1.9 ± 3.0	3.6 ± 5.0	<0.001

Multivariate regression analysis demonstrated that an mFI-5 score of 2 or more was an independent risk factor for wound complications [odds ratio (OR), 1.26; 95% confidence interval (CI), 1.08–2.20; *P* = 0.04] and overall complications (OR, 1.34; 95% CI, 1.09–2.15; *P* = 0.02) when controlling for potential confounding variables. BMI (OR, 1.05; 95% CI, 1.02–1.08; *P* < 0.001 and OR, 1.05; 95% CI, 1.03–1.08; *P* < 0.001) was also a significant independent risk factor for wound and overall complications, respectively (Table 4).

**Table 4.** Multivariate Regression Analysis of Wound and All Complications

Variable	Wound Complications			Complications		
	OR	95% CI	*P*	OR	95% CI	*P*
Frailty Index Score ≥2	1.26	1.08–2.20	0.04	1.34	1.09–2.15	0.02
BMI	1.05	1.02–1.08	<0.001	1.05	1.03–1.08	<0.001

Multivariate analysis also showed that BMI was an independent significant risk factor for longer hospital LOS (β = 0.15; 95% CI, 0.11–0.18; *P* < 0.001) (Table 5).

**Table 5.** Multivariate Regression Analysis of Hospital LOS

Variable	Hospital LOS		
	β	95% CI	*P*
Frailty Index Score	0.12	−0.52 to 0.76	0.71
BMI	0.15	0.11 to 0.18	<0.001

β, beta coefficient.

## DISCUSSION

The rising rate of obesity and bariatric surgery has led to an increase in the number of medically complex patients requesting body-contouring procedures.^15^ These post-bariatric patients can often have unique nutritional deficiencies that contribute to the risk of postoperative complications.^16^ A validated risk stratification tool can be a powerful adjunct to a surgeon when faced with the decision of whether or not surgery is appropriate in a widely heterogenous patient population.

The mFI-5 has been shown to be an independent predictor of postoperative complications in several surgical specialties,^11^ as well as a multitude of individual surgical procedures.^13,14,17–23^ The 5-item mFI is more practical to calculate as opposed to the 11-item mFI and the original 70-item index.^24,25^ Despite the decrease in input variables, the mFI-5 has been validated to correlate with the 11-item index as an independent predictor of postoperative complications in several surgical specialties.^11^ In a large retrospective study querying the NSQIP database, the mFI-5 was shown to hold a predictive value equal to that of the mFI-11 regarding mortality, postoperative complication, and unplanned 30-day readmission.^11^ We therefore sought to establish the validity of the mFI-5, a conceptually simple and holistic assessment of physiologic reserve, as a predictor for complications following panniculectomy.

There are several advantages of using the mFI-5 in preoperative risk stratification. The mFI-5 operationalizes the deficit accumulation approach to frailty, in which a patient’s frailty can be measured by the quantity of their health problems.^26^ The mFI-5 assesses the presence of diabetes mellitus, CHF, COPD, hypertension, and nonindependent functional status. Although many individual factors are known to contribute to the risk of postoperative complications following panniculectomy, assessing a patient’s cumulative frailty allows for a more comprehensive approach that better reflects the patient’s physiologic reserve compared with their peers. For example, age over 65 years has been shown to be an independent risk factor for overall complications following abdominal panniculectomy.^27^ Considering the patient population that should be risk stratified, only patients 65 years or older were included in this study, resulting in an average age of 68.8 years for the study population. Assessing frailty, as a cumulative decline in multiple physiological systems, allows for further risk stratification of an elderly patient population. Furthermore, frailty has been shown to be a superior predictor of morbidity and mortality than age.^28^

In the 575 patients included in this study, the overall complication rate of 23.3% and wound complication rate of 14.6% are comparable to those cited in the panniculectomy literature of 23%–44%.^4,5^ A threshold mFI-5 score of ≥2 was used based on the precedent set in previous studies assessing the predictive value of the mFI-5 for morbidity and mortality in various surgical subspecialties.^14,16,17,19,23^ In this study, patients with increased frailty were shown to have significantly higher rates of both wound and overall complications, as well as increased hospital LOS within 30 days following panniculectomy. This corresponds with the fact that several components of the mFI-5 are known individual risk factors for complications following abdominoplasty. Specifically, the presence of diabetes mellitus,^27^ cardiac comorbidities (including CHF and hypertension),^29,30^ and pulmonary comorbidities (including COPD)^30^ have all independently been identified as risk factors for postoperative complications.

Increased mFI-5 also correlated with several previously established risk factors for increased morbidity and mortality following panniculectomy. Patients with increased mFI-5 were significantly more likely to have an ASA class >3, which has been identified as an independent risk factor for morbidity following panniculectomy.^31,32^ In addition, patients with increased mFI-5 scores were more likely to be men, which has been previously identified as an independent risk factor for complications after abdominoplasty.^29^

We have found that increased BMI both correlates with increased mFI-5 and functions as an independent risk factor for wound and overall complications, as well as increased hospital LOS. This further supports the external validity of the mFI-5 as a predictor of morbidity following panniculectomy. Several studies have shown that higher preoperative BMI is an independent risk factor for the development of postoperative complications following panniculectomy.^32–34^ The increased rate of postsurgical complications in obese patients is thought to arise from associated comorbidities, as well as physiologic changes, such as chronic low-level inflammation and impaired drug clearance.^35,36^ Although the relationship between frailty and BMI is unclear, there is new literature exploring the contribution of BMI to frailty and their prognostic values for perioperative planning following bariatric surgery.^37,38^ This study adds to the growing evidence that supports the validity of the mFI-5 as a valid risk stratification tool to predict morbidity and mortality following surgery.

The mFI-5 as a perioperative risk stratification tool can be used for quality improvement in plastic surgery by contributing to the domain of patient safety.^39^ However, the mFI-5 is only one measure of perioperative risk and must be weighed against the improvement in a patient’s health when deciding the appropriateness of surgical intervention. Patient-reported outcome measures in post-bariatric patients indicate a substantial improvement in the quality of life following body-contouring surgery.^40^ Understanding additional risks incurred due to increased frailty could better inform expectations for the recovery process and improve preoperative surgical counseling.

Using the NSQIP database allows for a robust, retrospectively collected sample data. However, there are also several limitations inherent to the NSQIP database. Data availability is limited to 30 days postoperatively, and as a result, the incidence of complications may be underestimated. Findings are also limited to the 713 hospitals that are enrolled in the NSQIP database, which may differ from hospitals that do not participate in national data collection programs. Although these results may not be reflective of the overall patient population nationally, this is the largest study applying the mFI-5 to panniculectomy procedures to date. Finally, this is a retrospective study of the collected data while the proposed utility of the mFI-5 in preoperative risk stratification would be prospective. A prospective registry can therefore be maintained to assess the utility of the mFI-5 in practice.

## CONCLUSIONS

This study querying the NSQIP database for patients undergoing panniculectomy shows that frailty, as measured by the mFI-5, holds predictive value regarding outcomes of wound complications and overall complications. As such, the mFI-5 can be used by the surgeon preoperatively to enhance risk stratification and identify high-risk patients before panniculectomy. Future studies evaluating the prospective application of the mFI-5 before panniculectomy and its ability to improve perioperative evaluation are needed.

## ACKNOWLEDGMENT

This study was supported by American Diabetes Association Pathway Award 1-16-ACE-08 to Dr. Ceradini and the National Institute of Health Clinical and Translational Science Awards at NYU.
